# Supplementary material for: Associations between SNPs in Intestinal Cholesterol Absorption and Endogenous Cholesterol Synthesis Genes with Cholesterol Metabolism
Source: Biomedicines. 2021 Oct 14;9(10):1475. doi: 10.3390/biomedicines9101475 (PMC8533139; doi:10.3390/biomedicines9101475)
Supplement: Supplementary file 1 [file biomedicines-09-01475-s001.zip › Biomedicines Supplemental Information_resubmission.pdf]

SUPPLEMENTAL INFORMATION:

Associations between SNPs in Intestinal Cholesterol Absorption and Endogenous Cholesterol Synthesis Genes with Cholesterol Metabolism

Maite M. Schroor [1,*,†], Fatma B.A. Mokhtar [1,*,†], Jogchum Plat [1] and Ronald P. Mensink [1]

[1] Department of Nutrition and Movement Sciences, NUTRIM School of Nutrition and Translational Research in Metabolism, Maastricht University, 6200 MD Maastricht, The Netherlands.

* Correspondence: fatma.mokhtar@maastrichtuniversity.nl; Tel.: +31 (0)43 3881313 (F.B.A.M.); maite.schroor@maastrichtuniversity.nl; Tel.: +31 (0)43 3884258 (M.M.S.).

† These authors contributed equally to this work.

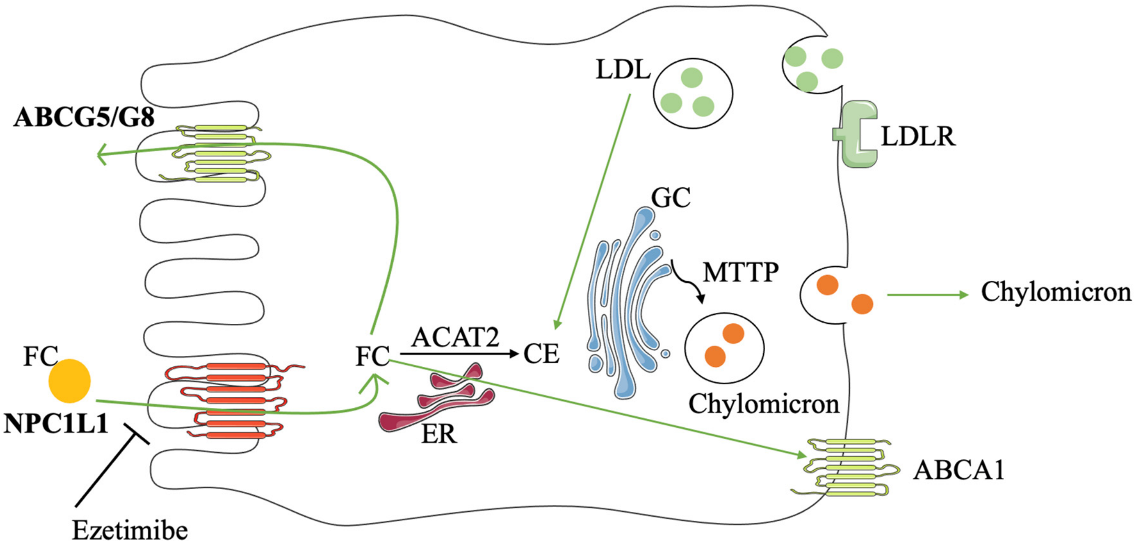

Figure S1. Schematic overview of the intestinal cholesterol absorption pathway. Free cholesterol (FC) enters the enterocyte via Niemann-Pick C1-like 1 (NPC1L1) and is esterified by Acetyl-CoA Acetyltransferase 2 (ACAT2) in the endoplasmic reticulum (ER). FC can also be transported back into the intestinal lumen via ATP-binding cassette member 5 and 8 (ABCG5/G8). Low-density lipoprotein (LDL) is taken up from the basolateral side via LDL receptor-mediated endocytosis. FC is also used by ATP-binding cassette A1 (ABCA1) to form high-density lipoprotein. Cholesterol ester (CE) is further processed in the Golgi complex (GC) with other components to form chylomicrons which are further transported to the lymphatic system. The drug ezetimibe is a NPC1L1 blocker and thus inhibits intestinal cholesterol absorption.

Note: Single-nucleotide polymorphisms in genes in bold have been included in the present study.

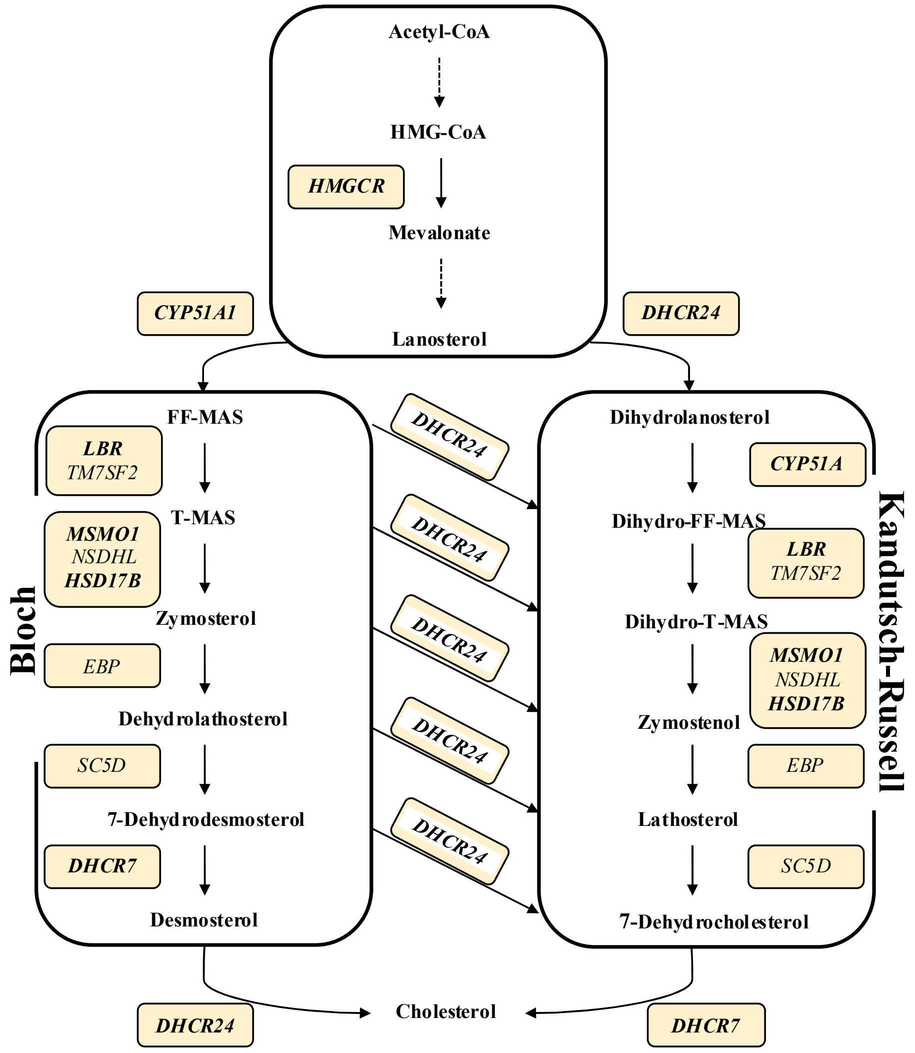

Figure S2. The endogenous cholesterol synthesis pathway. Cholesterol is synthesized via the Bloch and/or Kandutsch-Russell pathway. Similar enzymes are involved in these pathways, but intermediates differ.

Note: Single-nucleotide polymorphisms in genes in bold have been included in the present study.

Table S1. List of full names of genes included in the present study.

Gene symbol/ HGNC	Approved gene name in HGNC
Cholesterol absorption genes	
ABCG5	ATP binding cassette subfamily G member 5
ABCG8	ATP binding cassette subfamily G member8
NPC1L1	NPC1 like intracellular cholesterol transporter 1
Cholesterol synthesis genes	
CYP51A1	Cytochrome P450 family 51 subfamily A member 1
DHCR7	7-dehydrocholesterol reductase
DHCR24	24-dehydrocholesterol reductase
HMGCR	3 -hydroxy-3-methylglutaryl-CoA reductase
HSD17B7	Hydroxysteroid 17-beta dehyrogenase 7
LBR	Lamin B receptor
MSMO1	Methylsterol monooxygenase 1

Abbreviation: HGNC = Human Genome Organisation (HUGO) Gene Nomenclature Committee.

Table S2. Information given by the Precision Medicine Research Array for the two SNPs in *ABCG8* with an unknown rs-number.

Affymetrix SNP ID	Transcript ID Ensembl	SNP Location	Accession Number Nucleotide Database NCBI	Gene		
				Full name	Abbreviation	NCBI Gene ID
AX_11180448	ENST00000 272286	Missense	NM_022437	ATP-binding cassette, sub-family G (WHITE), member 8	ABCG8	64241
AX_82902928	ENST00000 272286	Intron	NM_022437	ATP-binding cassette, sub-family G (WHITE), member 8	ABCG8	64241

Accession Number Nucleotide Database NCBI = reference sequence of mRNA which links to the nucleotide database of NCBI; **Affymetrix SNP ID** = a unique Affymetrix identifier for the SNP; **NCBI Gene ID** = ID for a specific gene provided by NCBI; **Transcript ID Ensembl** = an identifier for the transcripts in the Ensembl database.

Table S3. Baseline characteristics for all participants and stratified by study.

	All subjects	Study 1	Study 2	Study 3	Study 4	Study 5	P-value
Age (years)	45.4 ± 15.3	33.0 ± 14.9	31.2 ± 13.8	50.0 ± 11.9	53.0 ± 10.1	33.0 ± 12.2	<0.001
Body mass index (kg/m^2)*	25.1 ± 3.6	23.1 ± 2.9	22.8 ± 2.5	25.3 ± 3.0	26.3 ± 3.6	23.9 ± 2.8	<0.001
Underweight	7 (1.5)	3 (2.8)	1 (2.9)	0 (0.0)	3 (1.2)	0 (0.0)	
Normal weight	225 (49.3)	76 (70.4)	27 (79.4)	18 (46.2)	92 (35.8)	12 (66.7)	
Overweight	179 (39.3)	27 (25.0)	6 (17.6)	21 (53.8)	119 (46.3)	6 (33.3)	< 0.001
Obesity class I	28 (6.1)	2 (1.9)	0 (0.0)	0 (0.0)	26 (10.1)	0 (0.0)	
Obesity class II	6 (1.3)	0 (0.0)	0 (0.0)	0 (0.0)	6 (2.3)	0 (0.0)	
Obesity class III	0 (0.0)	0 (0.0)	0 (0.0)	0 (0.0)	0 (0.0)	0 (0.0)	
Females	245 (55.7)	69 (63.9)	23 (67.6)	23 (59.0)	128 (49.8)	11 (61.1)	0.064
Smoking**	55 (12.1)	21 (19.4)	N/A	4 (10.3)	30 (11.7)	0 (100)	<0.001
Lipids							
TC	5.50 ± 1.02	4.95 ± 0.79	4.97 ± 0.91	5.95 ± 0.84	5.73 ± 1.05	5.38 ± 0.83	<0.001
HDL-C	1.46 ± 0.41	1.60 ± 0.38	1.42 ± 0.34	1.46 ± 0.44	1.40 ± 0.43	1.59 ± 0.33	<0.001
LDL-C	3.54 ± 0.95	2.93 ± 0.76	3.05 ± 0.89	3.93 ± 0.83	3.82 ± 0.91	3.20 ± 0.76	<0.001
TAG	0.97 (0.93 –	0.82 (0.75 –	0.99 (0.86 –	1.05 (0.88 –	1.00 (0.94 –	1.25 (1.06 –	<0.001
Non-cholesterol sterols***							
Lathosterol	107 ± 55	104 ± 35	131 ± 40	113 ± 54	101 ± 61	153 ± 70	<0.001
Sitosterol	137 ± 65	121 ± 44	153 ± 66	166 ± 54	136 ± 71	155 ± 60	<0.001
Campesterol	214 ± 120	321 ± 100	241 ± 126	256 ± 96	156 ± 95	249 ± 92	<0.001

Abbreviations: HDL-C = high-density lipoprotein cholesterol; LDL-C = low-density lipoprotein cholesterol; TAG = triacylglycerol; TC = total cholesterol.

Note: Categorical data are presented as n (%), and continuous data as mean ± SD. For TAG, the mean and (95% CI) are presented after back-transformation of the log-transformed values. Lipids are presented in mmol/L and the non-cholesterol sterols in 10^2 × µmol/mmol cholesterol.

* BMI data presented for N = 445, as data were unavailable for N = 11 in Study 4. BMI categories: underweight <18.5 kg/m^2, normal weight 18.5 – 24.9 kg/m^2, overweight 25.0 – 29.9 kg/m^2, obesity class I 30.0 – 34.9 kg/m^2, obesity class II 35.0 – 39.9 kg/m^2, and obesity class III ≥40 kg/m^2 [1].

** Smoking data presented for N = 410, because data were unavailable for N = 34 in S2 and for N = 12 in Study 4.

*** Non-cholesterol sterol levels presented for N = 455, because data were unavailable for N = 1 in Study 2.

Table S4. Associations between intestinal cholesterol absorption markers, an endogenous cholesterol synthesis marker and serum LDL-C concentrations.

Independent Variable	Dependent Variable	t	β	95 % CI for β		P-value
				Lower Bound	Upper Bound	
Sitosterol	Campesterol	42.424	1.39	1.321	1.449	**<0.001**
Sitosterol	Lathosterol	–2.253	–0.09	–0.169	–0.012	**0.025**
Campesterol	Lathosterol	–3.733	–0.10	–0.146	–0.045	**<0.001**
Campesterol	LDL-C	0.335	0.00	–0.001	0.001	0.738
Sitosterol	LDL-C	0.277	0.00	–0.001	0.001	0.782
Lathosterol	LDL-C	–0.403	0.00	–0.002	0.001	0.687

Abbreviations: LDL-C = low-density lipoprotein cholesterol

Note: Non-cholesterol sterols are presented in 10^2 × μmol/mmol cholesterol and LDL-C in mmol/L. All results were obtained from a linear regression analysis adjusted for the factor study.

Table S5. The location and allele frequencies for various SNPs in intestinal cholesterol absorption and endogenous cholesterol synthesis genes for 456 participants.

Gene	SNP	Location	Call rate (%)	Alleles	Frequencies		HWE
					Our cohort	European Cohort*	
	rs-number			(Ref/Alt)	(Ref/Alt)	(Ref/Alt)	P-value
Cholesterol absorption							
ABCG5	rs10208987	Intron	99.8	T/G	0.938/0.062	0.928/0.072	0.065
	rs4148189	Intron	100	C/T	0.899/0.101	0.888/0.112	0.741
	rs4245786	Intron	100	G/A	0.240/0.760	0.236/0.764	0.487
	rs7599296	Intron	100	G/A	0.813/0.188	0.832/0.168	0.751
	rs4148184	Intron	99.8	C/T	0.597/0.403	0.619/0.381	0.999
	rs13396273	Intron	100	C/T	0.649/0.351	0.640/0.360	0.518
ABCG8	AX_11180448**	Missense	100	G/C	0.932/0.068	-	0.033
	rs4148207	Intron	100	T/C	0.593/0.407	0.610/0.390	0.532
	rs4299376	Intron	98.2	G/T	0.320/0.680	0.323/0.677	0.004
	rs41360247	Intron	100	T/C	0.939/0.061	0.937/0.063	0.008
	rs6544713	Intron	100	T/C	0.310/0.690	0.322/0.678	0.019
	rs4245791	Intron	100	C/T	0.311/0.689	0.327/0.673	0.021
	rs13390041	Intron	100	A/G	0.543/0.457	0.554/0.446	0.694
	rs6709904	Intron	100	A/G	0.902/0.098	0.884/0.116	0.158
	rs4077440	Intron	99.8	T/C	0.442/0.558	0.439/0.561	0.511
	rs3795860	Intron	100	T/C	0.538/0.462	0.559/0.441	0.581
	AX_82902928**	Intron	100	AC/-	0.356/0.644	-	0.089
	rs55924588	Intron	100	T/C	0.950/0.050	0.935/0.065	0.257

NPC1L1	rs217429	Intron	100	A/C	0.754/0.246	0.751/0.249	0.898
	rs217416	Intron	99.6	T/C	0.736/0.264	0.737/0.263	0.105
	rs11763759	Intron	99.6	T/C	0.683/0.317	0.697/0.303	0.562
	rs2072183	Synonymous	99.8	G/C	0.769/0.231	0.774/0.226	0.100
Cholesterol synthesis							
CYP51A1	rs35968894	Intron	100	A/G	0.599/0.401	0.626/0.374	0.634
DHCR7	rs1792275	Intron	99.8	C/T	0.053/0.947	0.054/0.946	0.235
	rs72954301	Upstream	100	G/T	0.894/0.106	0.913/0.087	0.288
DHCR24	rs77668549	Intron	99.8	A/G	0.866/0.134	0.884/0.116	0.379
	rs7553385	Intron	100	A/G	0.938/0.062	0.942/0.058	0.300
	rs7551288	Intron	99.8	A/G	0.403/0.597	0.430/0.570	0.243
	rs11206456	Intron	100	C/T	0.917/0.083	0.905/0.095	0.512
	rs111480286	Intron	100	ACAG/-	0.934/0.066	0.941/0.059	0.434
	rs6676774	Intron	100	G/A	0.607/0.393	0.609/0.391	0.865
	rs718265	Synonymous	100	A/G	0.303/0.697	0.310/0.690	0.784
HMGCR	rs12654264	Intron	100	A/T	0.620/0.380	0.617/0.383	0.232
	rs3846662	Intron	100	A/G	0.553/0.447	0.564/0.436	0.319
	rs3846663	Intron	99.8	C/T	0.619/0.381	0.618/0.382	0.155
	rs12916	Prime UTR	100	T/C	0.587/0.413	0.594/0.06	0.342
HSD17B7	rs77482353	Intron	99.1	A/G	0.597/0.403	0.649/0.351	0.302
LBR	rs6678087	Intron	99.8	T/C	0.163/0.837	0.585/0.415	0.868
	rs12141732	Intron	99.8	T/C	0.712/0.288	0.700/0.300	0.395
	rs4653635	Intron	100	A/G	0.163/0.837	0.151/0.849	0.688
	rs12410357	Intron	100	G/A	0.889/0.111	0.875/0.125	0.449
MSMO1	rs17585739	Synonymous	100	G/A	0.944/0.056	0.938/0.062	0.611
	rs17046216	Intron	100	T/A	0.658/0.342	0.671/0.329	0.939

Abbreviations*:* Alt = Alternative allele; HWE = Hardy-Weinberg Equilibrium; Ref = Reference allele; SNP = single-nucleotide polymorphism.

* European cohort data (release version: 20201027095038) were obtained on January the 28th, 2021 from NCBI [2]. *HSD17B7* (rs7748253) was merged into rs11590043 on July the 1st, 2015.

** SNPs with unknown rs-numbers and European cohort frequencies.

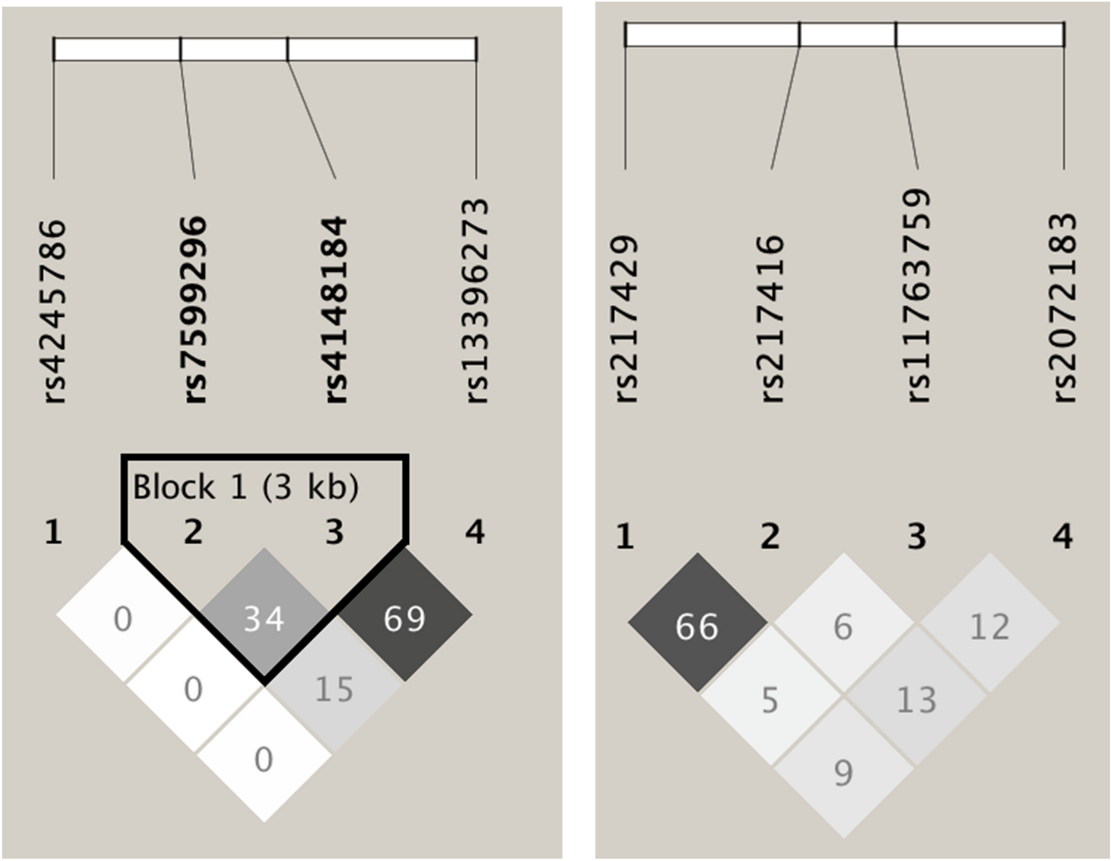

(a) (b)

Figure S3. Pairwise LD among SNPs in (a) *ABCG5* and (b) *NPC1L1* is indicated in the diamond shapes. The triangle marks the haplotype block within each region (based on the confidence interval of D′). The shading with a dark grey to white gradient indicates higher to lower LD between each pair of SNPs based on the r^2-value The LD plots were created by Haploview version 4.1 [3].

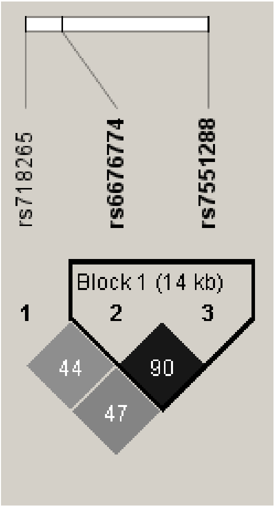

(a)

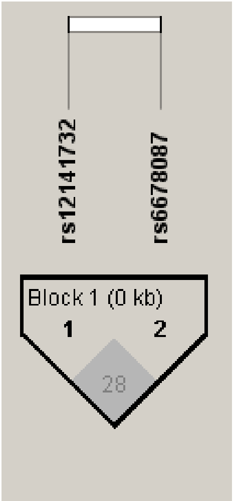

(b)

Figure S4. Pairwise LD among SNPs in (a) *DHCR24*, and (b) *LBR* is indicated in the diamond shapes. The triangle marks the haplotype block within each region (based on the confidence interval of D′). The shading with a dark grey to white gradient indicates higher to lower LD between each pair of SNPs based on the r^2-value The LD plots were created by Haploview version 4.1 [3].

Table S6. Associations between various SNPs in cholesterol absorption genes, that were either captured by a tag SNP or contained a genotype group < 12 individuals, with serum TC-standardized campesterol, sitosterol and lathosterol levels (N = 455), and serum LDL-C concentrations (N = 456).

Gene	SNP	Genotype	N	Campesterol 10²×µmol/mmol TC		Sitosterol 10²×µmol/mmol TC		Lathosterol 10²×µmol/mmol TC		N	LDL-C mmol/l	
				Mean (95% CI)	P-value	Mean (95% CI)	P-value	Mean (95% CI)	P-value		Mean (95% CI)	P-value
ABCG5	rs10208987	TT	403	249 (235 – 263)		149 (140 – 158)^{A}		119 (111 – 127)		403	3.39 (3.27 – 3.51)	
		TG	47	214 (184 – 244)	**0.041**	124 (104 – 143)^{B}	**0.018**	131 (115 – 148)	0.340	48	3.38 (3.12 – 3.64)	0.900
		GG	4	195 (98 – 292)		112 (50 – 175)		124 (70 – 178)		4	3.19 (2.33 – 4.05)	
	rs4148189	TT	4	290 (192 – 388)		158 (94 – 221)		114 (60 – 167)		4	2.94 (2.08 – 3.80)	
		TC	83	227 (204 – 251)	0.137	136 (121 – 151)	0.236	137 (125 – 150)^{A}	**0.005**	84	3.43 (3.22 – 3.63)	0.533
		CC	368	249 (234 – 263)		149 (140 – 158)		116 (108 – 124)^{B}		368	3.38 (3.25 – 3.51)	
ABCG8	AX_11180448	CC	5	193 (106 – 280)		126 (70 – 182)		150 (102 – 198)		5	3.05 (2.28 – 3.81)	
		CG	51	217 (189 – 245)	**0.040**	125 (107 – 143)^{A}	**0.022**	117 (101 – 132)	0.427	52	3.28 (3.03 – 3.52)	0.381
		GG	399	250 (236 – 264)		150 (141 – 159)^{B}		121 (113 – 129)		399	3.41 (3.29 – 3.54)	
	rs4299376*	TT	194	220 (204 – 237)^{A}		130 (119 – 140)^{A}		124 (114 – 133)		194	3.34 (3.19 – 3.49)	
		TG	220	256 (239 – 272)^{B}	**<0.001$^{\$}$**	153 (143 – 164)^{B}	**<0.001$^{\$}$**	118 (109 – 128)	0.577	221	3.41 (3.27 – 3.56)	0.662
		GG	33	320 (286 – 254)^{C}		201 (179 – 222)^{C}		117 (98 – 137)		33	3.36 (3.05 – 3.67)	
	rs41360247	TT	405	249 (235 – 264)		150 (141 – 159)		120 (113 – 128)		405	3.42 (3.29 – 3.54)	
		TC	45	216 (187 – 246)	0.052	124 (105 – 144)	**0.031**	119 (102 – 135)	0.468	46	3.22 (2.97 – 3.48)	0.239
		CC	5	193 (106 – 280)		126 (70 – 182)		150 (102 – 198)		5	3.05 (2.28 – 3.81)	
	rs6544713*	TT	33	316 (282 – 350)^{A}		198 (176 – 220)^{A}		117 (98 – 137)		33	3.38 (3.08 – 3.69)	
		TC	216	256 (239 – 272)^{B}	**<0.001$^{\$}$**	153 (143 – 164)^{B}	**<0.001$^{\$}$**	118 (109 – 128)	0.653	217	3.45 (3.31 – 3.60)	0.285
		CC	206	221 (205 – 237)^{C}		130 (120 – 141)^{C}		123 (114 – 132)		206	3.32 (3.17 – 3.47)	
	rs6709904	AA	374	246 (231 – 260)		147 (137 – 156)		121 (113 – 129)		374	3.41 (3.28 – 3.54)	
		AG	74	243 (219 – 268)	0.443	145 (130 – 161)	0.948	116 (103 – 129)	0.576	75	3.33 (3.21 – 3.54)	0.306
		GG	7	198 (124 – 272)		139 (91 – 187)		135 (95 – 176)		7	2.94 (2.29 – 3.59)	
	rs55924588	TT	409	247 (232 – 261)		147 (138 – 156)		121 (113 – 128)		410	3.38 (3.26 – 3.50)	
		TC	46	223 (192 – 254)	0.124	136 (116 – 156)	0.250	117 (100 – 134)	0.676	46	3.45 (3.18 – 3.71)	0.632
		CC	0	N/A		N/A		N/A		0	N/A	
	rs13390041*	AA	131	252 (232 – 272)		153 (140 – 166)		120 (109 – 131)		132	3.33 (3.15 – 3.50)	
		AG	213	245 (229 – 261)	0.378	146 (136 – 157)	0.168	123 (114 – 132)	0.527	231	3.46 (3.32 – 3.60)	0.180
		GG	93	233 (210 – 256)		136 (122 – 151)		115 (103 – 128)		93	3.29 (3.09 – 3.49)	

Abbreviations: LDL-C = low-density lipoprotein cholesterol; N/A = not applicable; SNP = single-nucleotide polymorphism; TC = total cholesterol.

Note: All analyses were adjusted for the factor study. Data are presented as estimated marginal means (95% CI). Non-cholesterol sterol levels were missing for N = 1. Different letters within a SNP indicate significantly different non-cholesterol sterol levels between the genotypes based on a Bonferroni post-hoc test.

* Indicates a SNP captured by a tag SNP. $ Additive models are presented in the supplemental material (**Table S7**).

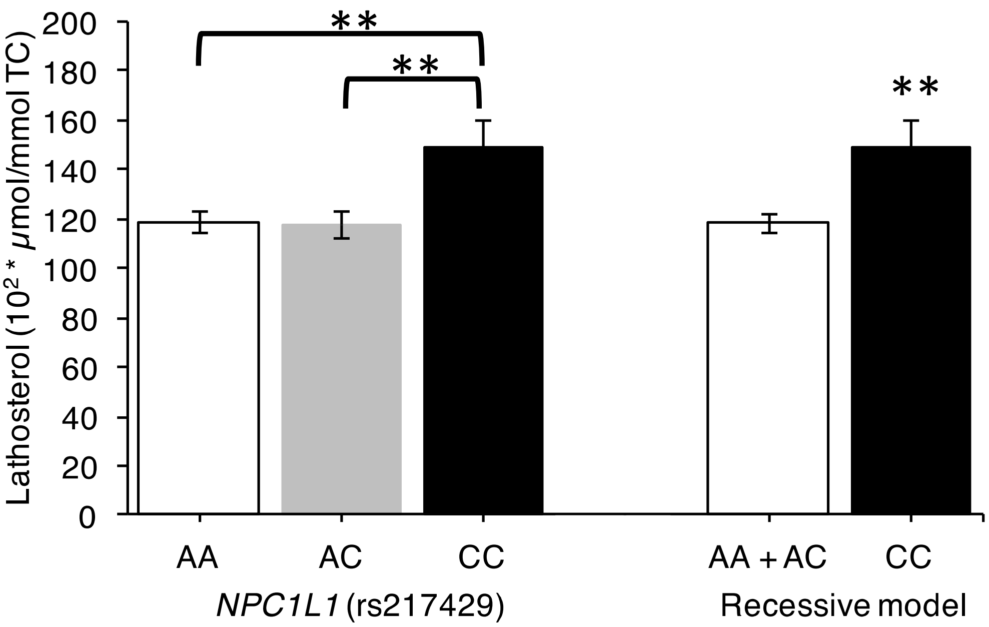

(a)

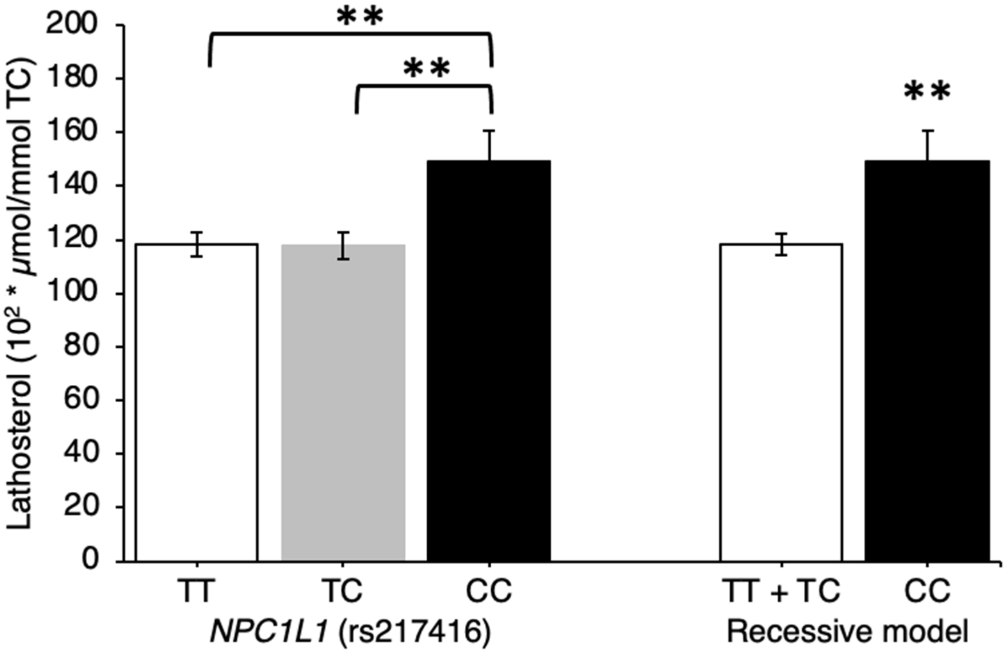

(b)

Figure S5. Association between SNPs (a) *NPC1L1* (rs217429) and (b) *NPC1L1* (rs217416) with serum levels of cholesterol-standardized lathosterol using recessive models. All values were adjusted for the factor study and presented as estimated marginal means ± SE. The black bars

refer to the least frequent homozygous genotype, and the white bars refer to the most frequent homozygous genotype. * $p \leq 0.05$, ** $p \leq 0.01$.

Table S7. Associations between SNPs in intestinal cholesterol absorption genes with TC-standardized non-cholesterol sterols using additive models (N = 455).

**Gene**	**SNP**	**Alleles (Ref/Alt)**	**Marker**	**t**	**β**	**95 % CI for β**		**P-value**
						Lower Bound	Upper Bound	
ABCG5	rs4245786	G/A	Sitosterol	-1.4	-6.9	-16.5	2.7	0.161
ABCG8	rs6544713	T/C	Campesterol	5.7	41.5	27.3	55.8	**<0.001**
ABCG8	rs6544713	T/C	Sitosterol	6.2	28.7	19.6	37.9	**<0.001**
ABCG8	rs4245791	C/T	Campesterol	5.8	41.4	27.2	55.5	**<0.001**
ABCG8	rs4245791	C/T	Sitosterol	6.2	28.6	19.5	37.7	**<0.001**
ABCG8	rs4299376	G/T	Campesterol	5.8	42.9	28.5	75.4	**<0.001**
ABCG8	rs4299376	G/T	Sitosterol	6.3	29.7	20.4	38.9	**<0.001**

Abbreviations: Alt = alternative allele; Ref = reference allele; SNP= single-nucleotide polymorphism

Note: Non-cholesterol sterols are presented in $10^2 \times$ µmol/mmol total cholesterol. All results were obtained from a linear regression analysis adjusted for the factor study. The alternative allele was used as reference in the model; each copy of the reference allele changes the outcome parameter (marker) with β.

Table S8. Associations between various SNPs in genes involved in intestinal cholesterol absorption with serum total cholesterol concentrations (N = 456).

Gene	SNP	Genotype	N	Total cholesterol mmol/L	
				Mean (95% CI)	P-value
ABCG5	rs10208987	TT	403	5.40 (5.26 – 5.53)	
		TG	48	5.40 (5.11 – 5.69)	0.906
		GG	4	5.19 (4.24 – 6.14)	
	rs4148189	TT	4	5.13 (4.81 – 6.09)	
		TC	84	5.43 (5.21 – 5.66)	0.803
		CC	368	5.39 (5.25 – 5.53)	
	rs4245786	AA	266	5.45 (5.29 – 5.60)	
		AG	161	5.35 (5.18 – 5.53)	0.356
		GG	29	5.21 (4.85 – 5.58)	
	rs7599296	AA	15	5.16 (4.66 – 5.66)	
		AG	141	5.42 (5.23 – 5.60)	0.616
		GG	300	5.40 (5.26 – 5.55)	
	rs4148184	TT	74	5.22 (4.98 – 5.46)	
		TC	219	5.41 (5.24 – 5.57)	0.182
		CC	162	5.47 (5.29 – 5.65)	
	rs13396273	TT	53	5.25 (4.98 – 5.53)	
		TC	214	5.39 (5.23 – 5.55)	0.376
		CC	189	5.46 (5.29 – 5.63)	
ABCG8	AX_11180448	CC	5	5.16 (4.31 – 6.01)	
		CG	52	5.27 (5.00 – 5.54)	0.463
		GG	399	5.42 (5.29 – 5.56)	
	rs4148207	TT	157	5.43 (5.25 – 5.61)	
		TC	227	5.42 (5.26 – 5.58)	0.408
		CC	72	5.26 (5.02 – 5.50)	
	rs4299376	TT	194	5.33 (5.16 – 5.50)	
		TG	221	5.42 (5.25 – 5.58)	0.467
		GG	33	5.52 (5.18 – 5.86)	
	rs41360247	TT	405	5.44 (5.30 – 5.57)	
		TC	46	5.18 (4.90 – 5.47)	0.203
		CC	5	5.16 (4.31 – 6.01)	
	rs6544713	TT	33	5.54 (5.21 – 5.88)	
		TC	217	5.46 (5.30 – 5.63)	0.151
		CC	206	5.30 (5.14 – 5.47)	
	rs4245791	TT	206	5.31 (5.14 – 5.47)	
		TC	216	5.47 (5.31 – 5.63)	0.163
		CC	34	5.51 (5.18 – 5.85)	
	rs13390041	AA	132	5.41 (5.22 – 5.60)	
		AG	231	5.46 (5.30 – 5.61)	0.115
		GG	93	5.21 (4.99 – 5.44)	
	rs6709904	AA	374	5.42 (5.28 – 5.56)	
		AG	75	5.33 (5.10 – 5.56)	0.392
		GG	7	4.99 (4.27 – 5.71)	
	rs4077440	TT	92	5.49 (5.27 – 7.71)	
		TC	218	5.46 (5.30 – 5.62)	0.066
		CC	145	5.25 (5.06 – 5.43)	
	rs3795860	TT	129	5.41 (5.22 – 5.60)	
		TC	233	5.46 (5.30 – 5.61)	0.127
		CC	94	5.21 (5.00 – 5.44)	
	AX_82902928	--	197	5.45 (5.29 – 5.62)	
		-AC	193	5.41 (5.24 – 5.58)	0.057
		ACAC	66	5.13 (4.88 – 5.39)	
	rs55924588	TT	410	5.39 (5.26 – 5.53)	
		TC	46	5.46 (5.16 – 5.76)	0.645

		CC	0	N/A	
NPC1L1	rs217429	AA	259	5.39 (5.24 – 5.54)	
		AC	170	5.41 (5.23 – 5.59)	0.938
		CC	27	5.45 (5.08 – 5.83)	
	rs217416	TT	239	5.43 (5.28 – 5.59)	
		TC	190	5.36 (5.18 – 5.53)	0.698
		CC	25	5.37 (4.90 – 5.76)	
	rs11763759	TT	209	5.42 (5.26 – 5.58)	
		TC	202	5.31 (5.15 – 5.48)	**0.043**
		CC	43	5.71 (5.41 – 6.01)	
	rs2072183	CC	18	5.34 (4.88 – 5.80)	
		CG	174	5.41 (5.23 – 5.59)	0.956
		GG	263	5.40 (5.25 – 5.54)	

Abbreviations: N/A: not applicable; SNP = single-nucleotide polymorphism.

Note: All analyses were adjusted for the factor study. Data are presented as estimated marginal means (95% CI). Statistical significance was set a a p-value < 0.05.

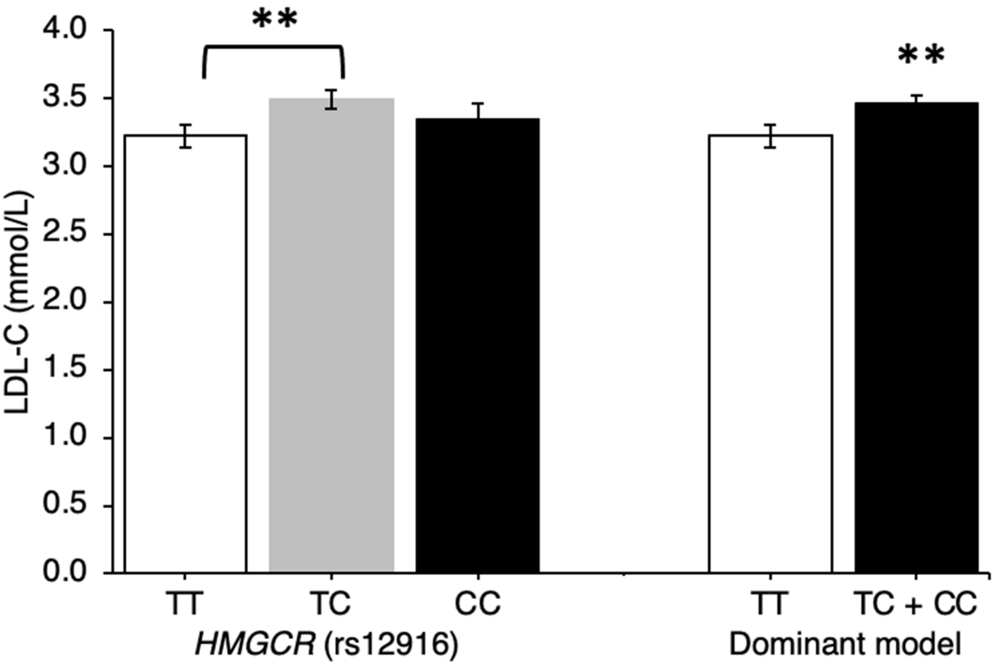

(a)

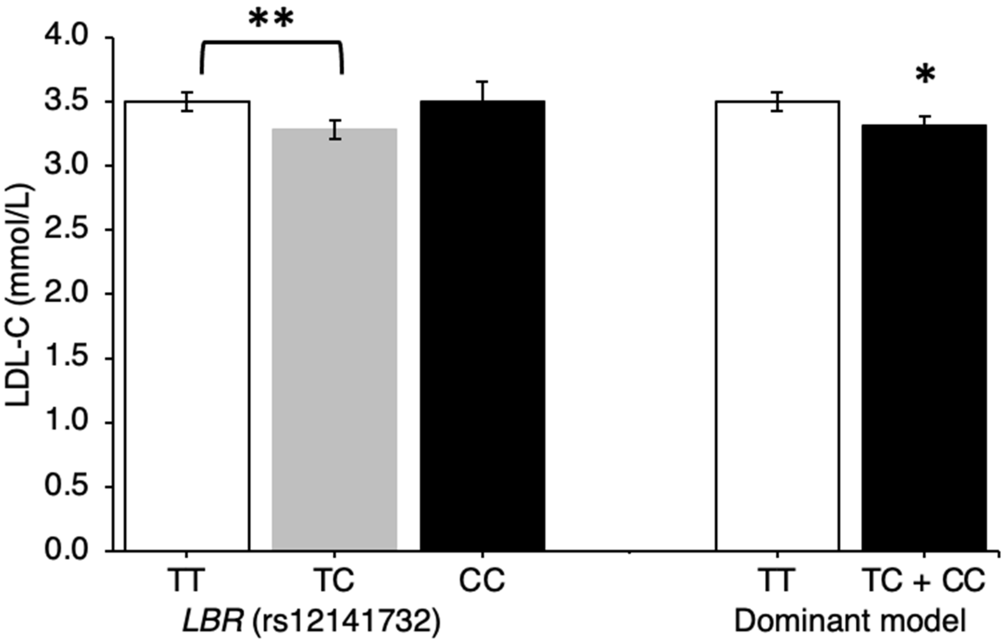

(b)

Figure S6. Association between SNPs (**a**) *HMGCR* (rs12916) and (**e**) *LBR* (rs12141732) with serum LDL-C concentrations using dominant models. All values were adjusted for the factor study and presented as estimated marginal means ± SE. The black bars refer to the least

frequent homozygous genotype, and the white bars refer to the most frequent homozygous genotype. * $p \leq 0.05$, ** $p \leq 0.01$.

Table S9. Associations between various SNPs in endogenous cholesterol synthesis genes, that were either captured by a tag SNP or contained a genotype group < 12 individuals, with serum TC-standardized campesterol, sitosterol and lathosterol levels (N = 455), and serum LDL-C concentrations (N = 456).

Gene	SNP	Genotype	N	Campesterol 10^2×µmol/mmol TC		Sitosterol 10^2×µmol/mmol TC		Lathosterol 10^2×µmol/mmol TC		N	LDL-C mmol/L	
				Mean (95% CI)	P-value	Mean (95% CI)	P-value	Mean (95% CI)	P-value		Mean (95% CI)	P-value
DHCR7	rs1792275	TT	406	243 (229 – 256)		145 (136 – 154)		123 (115 – 130)		407	3.37 (3.24 – 3.49)	
		TC	48	259 (230 – 289)	0.266	156 (137 – 175)	0.250	104 (88 – 120)	**0.024**	48	3.60 (3.34 – 3.86)	0.078
		CC	0	N/A		N/A		N/A		0	N/A	
	rs72954301	TT	3	207 (94 – 320)		121 (48 – 194)		137 (75 – 200)		3	3.35 (2.36 – 4.34)	
		TG	91	253 (230 – 276)	0.537	148 (133 – 163)	0.763	122 (109 – 135)	0.828	91	3.34 (3.14 – 3.54)	0.837
		GG	361	243 (229 – 258)		146 (137 – 155)		120 (112 – 128)		362	3.40 (3.27 – 3.52)	
DHCR24	rs77668549	AA	339	244 (229 – 258)		145 (136 – 155)		119 (111 – 127)		339	3.39 (3.26 – 3.52)	
		AG	109	252 (230 – 273)	0.354	152 (138 – 166)	0.461	125 (113 – 137)	0.654	110	3.36 (3.17 – 3.55)	0.765
		GG	6	194 (115 – 274)		124 (72 – 176)		134 (90 – 178)		6	3.62 (2.93 – 4.32)	
	rs7551288*	AA	80	232 (208 – 256)		144 (128 – 159)		119 (106 – 133)		80	3.40 (3.19 – 3.61)	
		AG	207	251 (234 – 267)	0.376	148 (138 – 159)	0.799	123 (114 – 133)	0.519	207	3.33 (3.18 – 3.48)	0.358
		GG	167	244 (225 – 262)		145 (133 – 157)		117 (107 – 127)		168	3.46 (3.30 – 3.62)	
	rs7553385	AA	401	246 (232 – 250)		147 (138 – 156)		121 (113 – 128)		402	3.39 (3.26 – 3.51)	
		AG	51	229 (200 – 259)	0.494	139 (121 – 158)	0.725	119 (103 – 135)	0.916	51	3.34 (3.09 – 3.60)	0.211
		GG	3	261 (149 – 374)		144 (72 – 217)		109 (46 – 171)		3	4.25 (3.26 – 5.23)	
	rs11206456	TT	2	224 (86 – 362)		137 (48 – 226)		140 (64 – 216)		2	3.88 (2.67 – 5.09)	
		TC	72	255 (230 – 280)	0.608	153 (137 – 170)	0.605	129 (115 – 143)	0.323	72	3.33 (3.11 – 3.55)	0.597
		CC	381	243 (229 – 257)		145 (136 – 154)		119 (111 – 127)		382	3.40 (3.27 – 3.52)	
	rs111480286	--	3	261 (149 – 374)		145 (72 – 217)		109 (46 – 171)		3	4.25 (3.26 – 5.23)	
		-ACAG	54	230 (202 – 259)	0.512	140 (121 – 158)	0.730	118 (103 – 134)	0.891	54	3.29 (3.04 – 5.53)	0.153
		ACAGACAG	398	246 (232 – 260)		147 (138 – 156)		121 (113 – 129)		399	3.39 (3.27 – 3.52)	
	rs4653635	AA	11	244 (185 – 304)		148 (110 – 187)		100 (68 – 133)		11	3.05 (2.53 – 3.57)	
		AG	127	235 (214 – 255)	0.426	139 (126 – 152)	0.351	121 (109 – 132)	0.496	127	3.46 (3.28 – 3.63)	0.275
		GG	317	248 (233 – 263)		149 (139 – 158)		121 (113 – 129)		318	3.37 (3.24 – 3.50)	
	rs12410357	AA	4	284 (186 – 382)		138 (75 – 202)		125 (71 – 179)		4	4.10 (3.24 – 4.95)	
		AG	93	244 (221 – 267)	0.728	139 (124 – 154)	0.450	125 (112 – 138)	0.690	93	3.34 (3.13 – 3.54)	0.219
		GG	358	245 (230 – 259)		148 (139 – 157)		119 (112 – 127)		359	3.39 (3.27 – 3.52)	
HMGCR	rs12654264*	AA	168	240 (221 – 259)	0.699	144 (132 – 157)	0.906	112 (112 – 133)	0.820	169	3.24 (3.08 – 3.40)[A]	**0.021**
		AT	227	245 (229 – 262)		147 (137 – 158)		119 (110 – 128)		227	3.48 (3.34 – 3.62)[B]	

		TT	60	253 (226 – 279)		147 (130 – 165)		122 (107 – 136)		60	3.40 (3.17 – 3.63)	
	rs3846662*	AA	134	239 (218 – 259)		144 (131 – 157)		117 (106 – 129)		134	3.20 (3.02 – 3.38)[A]	
		AG	235	243 (227 – 258)	0.339	145 (134 – 155)	0.426	121 (112 – 130)	0.717	236	3.46 (3.32 – 3.59)[B]	**0.020**
		GG	86	258 (235 – 281)		154 (140 – 169)		123 (110 – 136)		86	3.43 (3.23 – 3.63)	
	rs3846663*	TT	59	245 (218 – 272)		143 (126 – 160)		122 (107 – 137)		59	3.39 (3.16 – 3.63)	
		TC	229	244 (228 – 260)	0.811	146 (136 – 157)	0.905	119 (110 – 128)	0.809	229	3.48 (3.34 – 3.62)[A]	**0.018**
		CC	166	238 (220 – 257)		144 (132 – 156)		122 (112 – 133)		167	3.23 (3.07 – 3.94)[B]	
LBR	rs4653635	AA	11	244 (185 – 304)		148 (110 – 187)		100 (68 – 133)		11	3.05 (2.53 – 3.57)	
		AG	127	235 (214 – 255)	0.426	139 (126 – 152)	0.351	121 (109 – 132)	0.496	127	3.46 (3.28 – 3.63)	0.275
		GG	317	248 (233 – 263)		149 (139 – 158)		121 (113 – 129)		318	3.37 (3.24 – 3.50)	
	rs12410357	AA	4	284 (186 – 382)		138 (75 – 202)		125 (71 – 179)		4	4.10 (3.24 – 4.95)	
		AG	93	244 (221 – 267)	0.728	139 (124 – 154)	0.450	125 (112 – 138)	0.690	93	3.34 (3.13 – 3.54)	0.219
		GG	358	245 (230 – 259)		148 (139 – 157)		119 (112 – 127)		359	3.39 (3.27 – 3.52)	
MSMO1	rs17585739	AA	2	231 (91 – 371)		140 (50 – 231)		91 (14 – 168)		2	3.23 (2.01 – 4.46)	
		AT	47	238 (231 – 260)	0.865	140 (120 – 159)	0.759	125 (109 – 142)	0.638	47	3.40 (3.14 – 3.67)	0.965
		TT	406	246 (231 – 250)		147 (138 – 156)		120 (112 – 128)		407	3.39 (3.26 – 3.51)	

Abbreviations: LDL-C = low-density lipoprotein cholesterol; N/A = not applicable; SNP = single-nucleotide polymorphism; TC = total cholesterol.

Note: All analyses were adjusted for the factor study. Data are presented as estimated marginal means (95% CI). Non-cholesterol sterol levels were missing for N = 1. Different letters within a SNP indicate significantly different non-cholesterol sterol levels or LDL-C concentrations between the genotypes based on a Bonferroni post-hoc test. * Indicates a SNP captured by a tag SNP.

Table S10. Associations between various SNPs in genes involved in endogenous cholesterol synthesis with serum total cholesterol concentrations (N = 456).

Gene	SNP	Genotype	N	Total cholesterol mmol/L Mean (95% CI)	P-value
CYP51A1	rs35968894	AA	161	5.40 (5.22 – 5.57)	0.993
		AG	224	5.39 (5.23 – 5.56)	
		GG	71	5.41 (5.17 – 5.65)	
DHCR7	rs1792275	TT	407	5.37 (5.24 – 5.51)	0.075
		TC	48	5.63 (5.35 – 5.92)	
		CC	0	N/A	
	rs72954301	TT	3	5.50 (4.40 – 6.59)	0.453
		TG	91	5.28 (5.06 – 5.51)	
		GG	362	5.43 (5.29 – 5.56)	
DHCR24	rs77668549	AA	339	5.41 (5.27 – 5.55)	0.738
		AG	110	5.36 (5.15 – 5.57)	
		GG	6	5.64 (4.87 – 6.41)	
	rs7553385	AA	402	5.40 (5.26 – 5.53)	0.347
		AG	51	5.38 (5.09 – 5.66)	
		GG	3	6.20 (5.11 – 7.29)	
	rs7551288	AA	80	5.44 (5.21 – 5.67)	0.700
		AG	207	5.36 (5.19 – 5.52)	
		GG	168	5.43 (5.25 – 5.61)	
	rs11206456	TT	2	5.81 (4.47 – 7.15)	0.725
		TC	72	5.34 (5.10 – 5.59)	
		CC	382	5.41 (5.27 – 5.54)	
	rs111480286	--	3	6.20 (5.11 – 7.29)	0.276
		-ACAG	54	5.31 (5.03 – 5.58)	
		ACAGACAG	399	5.40 (5.27 – 5.54)	
	rs6676774	AA	75	5.45 (5.21 – 5.68)	0.427
		AG	208	5.34 (5.18 – 5.50)	
		GG	173	5.46 (5.28 – 5.63)	
	rs718265	AA	43	5.30 (5.00 – 5.61)	0.437
		AG	190	5.36 (5.20 – 5.53)	
		GG	223	5.45 (5.29 – 5.62)	
HMGCR	rs12654264	AA	169	5.26 (5.08 – 5.44)[A]	0.037
		AT	227	5.51 (5.35 – 5.66)[B]	
		TT	60	5.34 (5.08 – 5.60)	
	rs3846662	AA	134	5.24 (5.04 – 5.44)	0.087
		AG	236	5.47 (5.31 – 5.62)	
		GG	86	5.41 (5.18 – 5.63)	
	rs3846663	TT	59	5.34 (5.08 – 5.60)[A]	0.034
		TC	229	5.51 (5.35 – 5.66)[B]	
		CC	167	5.26 (5.08 – 5.44)	
	rs12916	TT	152	5.24 (5.06 – 5.43)[A]	0.022
		TC	231	5.51 (5.36 – 5.67)[B]	
		CC	73	5.32 (5.08 – 5.55)	
HSD17B7	rs77482353	AA	156	5.39 (5.21 – 5.57)	0.103
		AG	228	5.34 (5.18 – 5.50)[A]	
		GG	68	5.62 (5.37 – 5.87)[B]	
LBR	rs6678087	TT	141	5.41 (5.22 – 5.60)	0.530
		TC	223	5.36 (5.20 – 5.52)	
		CC	91	5.49 (5.27 – 5.71)	
	rs12141732	TT	227	5.52 (5.36 – 5.44)[A]	0.032
		TC	194	5.28 (5.12 – 5.88)[B]	
		CC	34	5.54 (5.20 – 286)	

	rs4653635	AA	11	5.11 (4.53 – 5.69)	
		AG	127	5.41 (5.21 – 5.60)	0.600
		GG	318	5.40 (5.26 – 5.55)	
	rs12410357	AA	4	6.34 (5.39 – 7.29)	
		AG	93	5.33 (5.10 – 5.55)	0.111
		GG	359	5.41 (5.27 – 5.54)	
MSMO1	rs17585739	AA	2	5.54 (4.18 – 6.90)	
		AT	47	5.43 (5.13 – 5.72)	0.956
		TT	407	5.39 (5.26 – 5.53)	
	rs17046216	AA	53	5.68 (5.41 – 5.96)	
		AG	206	5.39 (5.23 – 5.55)	0.060
		GG	197	5.33 (5.17 – 5.50)	

Abbreviations: N/A: not applicable; SNP = single-nucleotide polymorphism.

Note: All analyses were adjusted for the factor study. Data are presented as estimated marginal means (95% CI). Different letters within a SNP indicate significantly different TC concentrations between the genotypes based on a Bonferroni post-hoc test. Statistical significance was set a a p-value < 0.05.

References

1. World Health Organization. Body mass index - BMI. Available online: https://www.euro.who.int/en/health-topics/disease-prevention/nutrition/a-healthy-lifestyle/body-mass-index-bmi].
2. Database of Single Nucleotide Polymorphisms (dbSNP). Bethesda (MD): National Center for Biotechnology Information, National Library of Medicine. dbSNP accession:(dbSNP Build ID: 154). Available online: http://www.ncbi.nlm.nih.gov/SNP/].
3. Barrett, J.C.; Fry, B.; Maller, J.; Daly, M.J. Haploview: analysis and visualization of LD and haplotype maps. *Bioinformatics* **2005**, *21*, 263-265, doi:10.1093/bioinformatics/bth457.
